# 3D Numerical Simulation and Structural Optimization for a MEMS Skin Friction Sensor in Hypersonic Flow

**DOI:** 10.3390/mi13091487

**Published:** 2022-09-07

**Authors:** Huihui Guo, Xiong Wang, Tingting Liu, Zhijiang Guo, Yang Gao

**Affiliations:** 1 School of Information Engineering, Southwest University of Science and Technology, Mianyang 621010, China; 2 Robot Technology Used for Special Environment Key Laboratory of Sichuan Province, Mianyang 621010, China; 3 Hypervelocity Aerodynamics Institute, China Aerodynamics Research and Development Center, Mianyang 621010, China

**Keywords:** skin friction sensor, 3D models, CFD, turbulent flow, laminar flow, friction measurement accuracy

## Abstract

The skin friction of a hypersonic vehicle surface can account for up to 50% of the total resistance, directly affecting the vehicle’s effective range and load. A wind tunnel experiment is an important and effective method to optimize the aerodynamic shape of aircraft, and Micro-Electromechanical System (MEMS) skin friction sensors are considered the promising sensors in hypersonic wind tunnel experiments, owing to their miniature size, high sensitivity, and stability. However, the sensitive structure including structural appearance, a gap with the package shell, and flatness of the sensor will change the measured flow field and cause the accurate measurement of friction resistance. Aiming at the influence of sensor-sensitive structure on wall-flow characteristics and friction measurement accuracy, the two-dimensional and three-dimensional numerical models of the sensor in the hypersonic flow field based on Computational Fluid Dynamics (CFD) are presented respectively in this work. The model of the sensor is verified by using the Blathius solution of two-dimensional laminar flow on a flat plate. The results show that the sensor model is in good agreement with the Blathius solution, and the error is less than 0.4%. Then, the influence rules of the sensitive structure of the sensor on friction measurement accuracy under turbulent flow and laminar flow conditions are systematically analyzed using 3D numerical models of the sensor, respectively. Finally, the sensor-sensitive unit structure’s design criterion is obtained to improve skin friction’s measurement accuracy.

## 1. Introduction

The skin friction of an aircraft surface is the main part of its total resistance, which can account for up to 50% and greatly limits the effective range and load of hypersonic vehicles. A wind tunnel experiment is an important way and effective method to optimize the aerodynamic shape of aircraft. Therefore, the skin friction measurement of the aircraft model is an important basic item in wind tunnel experiment research. In the hypersonic wind tunnel experiment, obtaining the accurate friction distribution of the model points or surfaces puts forward higher requirements for the size of the sensor limited by the volume of the aircraft model. In recent years, several types of MEMS sensors have been reported to measure skin friction including capacitance type and comb differential capacitance type [1,2,3], piezoresistive type [4,5,6], and piezoelectric type [7,8]. Those MEMS sensors have similar characteristics such as small size, high sensitivity, and high resolution in a small measurement range (several Pa).

Owing to their miniature size, high sensitivity, and stability, the MEMS skin friction sensors are considered the promising sensors in wind tunnel experiments. For example, Jiang et al. [1] have reported a supporting cantilever and plate differential capacitive skin friction sensor for low-speed wind tunnels with high sensitivity and a minimum detection limit of 0.05 Pa in a range from 0.1–2 Pa. Von P et al. [7] have reported a wall shear stress sensor using four piezoresistors as a transducer in the cantilever, with a resolution of 0.01 Pa in the range 0–2 Pa. T Kim T et al. [9] have reported a piezoelectric floating element shear stress sensor for the wind tunnel flow measurement with the high sensitivity 56.5 pc/Pa. However, these sensors are mainly used for skin friction detection in low wind tunnels because their sensing element such as comb capacitance was exposed in the flow field.

With the development of supersonic aircraft, direct skin friction measurements under hypersonic conditions are strongly required in wind tunnel experiments. At present, the environment of the hypersonic wind tunnel is relatively harsh, such as heavy load, particle inclusion in gas, etc. It is difficult for various comb capacitive sensors to survive in the hypersonic wind tunnel. To adapt to the harsh measurement environment and large normal loads in hypersonic fields, a MEMS skin friction sensor has been developed in our previous work [3,10], which adopts the floating element even with the measured wall and the signal output micro-structure being isolated from the hypersonic field. The sensor is composed of a silicon differential-capacitor embedded with a floating element, signal readout circuit, and a package metal shell, which was fabricated by using various micro-mechanical processes and micro-assembly technology. Experiments result show that the MEMS has good linearity, small size, high sensitivity, and repeatability of static calibration, the resolution is 0.1 Pa in ranges from 0 to 100 Pa, and the repeatability accuracy and linearity are better than 1%. However, the deviations between the measured skin friction coefficients and the analytical values are close to 100% under laminar flow conditions [10]. The reason for the large measurement error under the condition of laminar flow is that the friction coefficient itself is very small, and the small flow state interference from the sensor structure will cause a large deviation.

Generally, the height of the vicious bottom layer of low-speed and low shear flow is usually greater than 100 microns, and the influence of sensor structure error (tens of microns) on the fluid-structure can be ignored; the fluid-structure is considered hydraulic smoothing [10,11]. However, in high-speed and high shear flow, especially laminar flow, the height of the vicious bottom layer may be in the order of microns. At this time, the fluid structure is not considered hydraulic smoothing, the structure error of the sensor including the protrusion of the sensing element and the measurement gap will change the characteristics of the fluid, which will seriously affect the accuracy of the sensor in measuring the skin friction resistance. Therefore, it is of great significance to study the influence of sensor structure on the high-speed flow field. In this work, the two-dimensional and three-dimensional numerical models of the sensor in plate model under the hypersonic flow field based on CFD are presented respectively to research the influence of sensor-sensitive structure on wall-flow characteristics and friction perceptual characteristics. In addition, to improve the measurement accuracy of skin friction, the design criterion of the sensor-sensitive unit structure should be obtained.

## 2. A 3D Flat Plate Model Embedded with a MEMS Sensor

**Figure 1:**
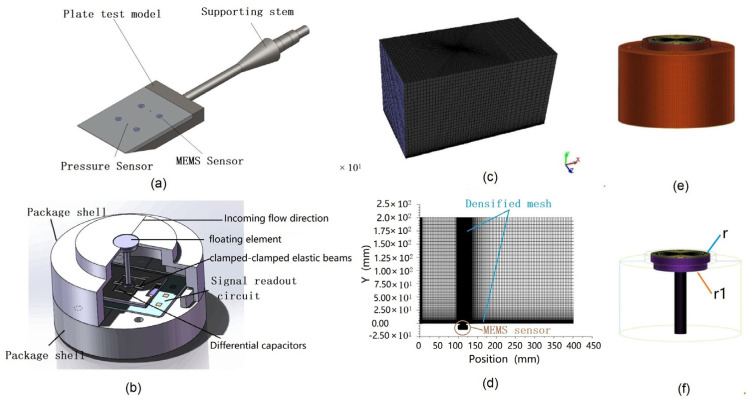
(**a**) Schematic of a flat plate model embedded with MEMS sensors: (**b**) schematic of the MEMS skin friction sensor; (**c**) the 3D mesh model of the flat plate model; (**d**) the lateral section of the 3D model as 2D plate model of the flat plate; (**e**) the 3D mesh model of the sensor; (**f**) the 3D mesh model of the floating element.

In the wind tunnel experiment, the measurement method of wall friction resistance is mainly based on the conventional friction balance and the friction measurement technology of the MEMS friction sensor, which is based on the plate model. The flat plate model is a classical and simple model, which is conducive to the installation of the sensor and the observation of the flow characteristics of the surface, and is also convenient for comparison with the Bratisius solution. The model of wind tunnel validation test is a flat plate model with tail support, and the MEMS sensors are installed in the inner cavity of the flat plate model, and the sensing surface of the measuring head is flush with the surface to be measured of the test model to directly feel the skin friction as shown in Figure 1a. The total length of the plate model is 400 mm and the width is 120 mm. The front sensor is 147 mm from the front edge of the plate model, and the rear sensor is 130 mm from the rear end face of the plate model. The two sides are symmetrically distributed along the neutral plane of the plate model, with a distance of 80 mm. The structure of MEMS skin friction is composed of a silicon differential-capacitor embedded with a floating element, signal readout circuit, and package metal shell as shown in Figure 1b. This paper focuses on the influence of the structure of the MEMS sensor on the measured flow field in hypersonic; therefore, except for the structure of the floating element, other structural parameters of the sensor remain unchanged. The structure parameters of the sensor are described in detail in our previous work [9]. The working principle of this sensor is explained as the skin friction causes the torsional deflection of clamped–clamped elastic beams by the floating element, and the torsional deflection is transferred into a capacitance variation by one pair of differential capacitors (as shown in Figure 1b). Finally, the friction coefficient is measured using the signal output circuit by measuring the change of the capacitors.

To research the influence of sensor-sensitive structure on wall-flow characteristics and friction perceptual characteristics, a 3D flat plate test model embedded with MEMS sensors is built by fluent which is a computer program used to simulate fluid flow and heat conduction with complex shapes, and the feature dimensions of the 3D model are 400 mm ×120 mm × 200 mm. The 2D plate model is the lateral section of the three-dimensional model, which is mainly used to compare with the Blasius solution to verify the effectiveness of the calculation model. In addition, the near-wall surface mesh of the numerical model needs to be densified to obtain more accurate numerical results, and the grid spacing of the first layer of the wall is 0.001 mm as shown in Figure 1d. The 3D model of the MEMS sensor is shown in Figure 1e, in which the diameters of the floating element with a two-layer structure are 5 mm (r) and 0.45 mm (r_1_) respectively, and the thickness is all 0.9 mm as shown in Figure 1f. Different from the previous structure [9], the floating element structure in this paper is large in the upper layer and small in the lower layer.

Combined with the flow field parameters of the wind tunnel test, the initial condition of the inner cavity of the friction sensor is set to the vacuum state, and the boundary conditions include wall boundary conditions, inflow boundary conditions and outflow boundary conditions are determined as follows: (1) the measured wall boundary meets the requirements of a no-slip wall (V = 0) for hypersonic viscous flow, and the wall temperature is constant temperature wall (T_w_ = 300 K); (2) inlet boundary of incoming flow is set as far-field input of pressure, the simulated incoming flow conditions are shown in Table 1; (3) outlet boundary selection pressure outlet boundary condition. Then, the numerical simulation of the 0° angle of attack attitude of the 2D plate model is calculated. The wind tunnel flow field parameters (such as dynamic pressure *q*_∞_, static pressure *P*_∞_, and unit Reynolds number *R_e_*/*L*) can be calculated according to the incoming total pressure *P*_0_, total temperature *T*_0_, and incoming Mach number *M*_∞_ as follows:

(1) $$P_{\infty }=P_{0}{\left(1+0.2M_{\infty }^{2}\right)}^{-3.5}$$

(2) $$T_{\infty }=T_{0}{\left(1+0.2M_{\infty }^{2}\right)}^{-1}$$

(3) $$q_{\infty }=0.7P_{\infty }M_{\infty }^{2}$$

(4) $$\mu_{\infty }=1.458\times 10^{-6}\frac{T_{\infty }^{1.5}}{T_{\infty }+110.4}$$

(5) $$R_{e}/L=\frac{0.05902M_{\infty }}{{\left(1+0.2M_{\infty }^{2}\right)}^{3}}\cdot \frac{P_{0}}{\mu_{\infty }}\cdot {\left(\frac{1.4}{T_{0}}\right)}^{0.5}$$

The calculation of friction coefficient (*C_f_*) is as follows:

(6) $$C_{f}=\frac{\tau_{\omega }}{{\;q}_{\infty }}$$

where $\tau_{w}$ is the measured skin friction, and *q*_∞_ is the dynamic pressure.

**Figure 2:**
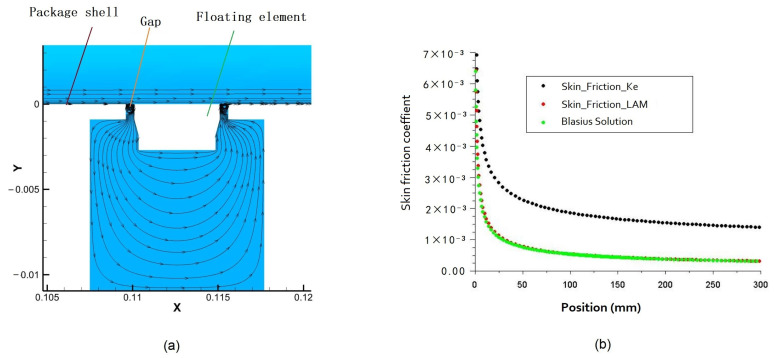
(**a**) The 2D streamline diagram in the friction sensor; (**b**) the skin friction coefficient of the flat plate in the laminar flow model and turbulent model under a total pressure of 1.5 MPa incoming flows.

The 3D calculation method is verified by using the Blasius solution of two-dimensional plate laminar flow under the condition of total incoming flow pressure of 1.5 MPa. In this work, the 2D plate model is the lateral section of the 3D model, which is mainly used to compare with the Blasius solution using the laminar flow model and turbulent model (k-e model). The 2D streamline diagram in the friction sensor is shown in Figure 2a, the X direction is the incoming flow direction, and the surface streamline shows that there is suction in the moving gap. It also can be seen that the airflow enters the cavity of the friction sensor along the wall from the gap inlet causing pressure resistance to the leading edge of the floating element head and affecting the friction coefficient. Meanwhile, the numerical simulation value of the friction coefficient at the installation position of the friction sensor (x = 112.6 mm) is 5.09 × 10^−4^ (directly read out in the model). Compared with Blasius solution 5.11 × 10^−4^ (Cf=0.667Rex), the difference is about 0.39%. Hence, the calculated results of plate surface friction coefficient are in good agreement with the Blasius solution using the laminar flow model as shown in Figure 2b, which also shows that the numerical simulation method and calculation model constructed in this paper is effective.

In addition, as shown in Figure 2b, it can also be seen that the friction coefficient in the laminar flow model state is smaller than that in the turbulent state, and the variation trend of the friction coefficient under the turbulent state is similar to that under laminar state, but the friction coefficient is larger than that under laminar state, and its value is around 0.0017.

## 3. Structural Optimization of the Floating Element

**Figure 3:**
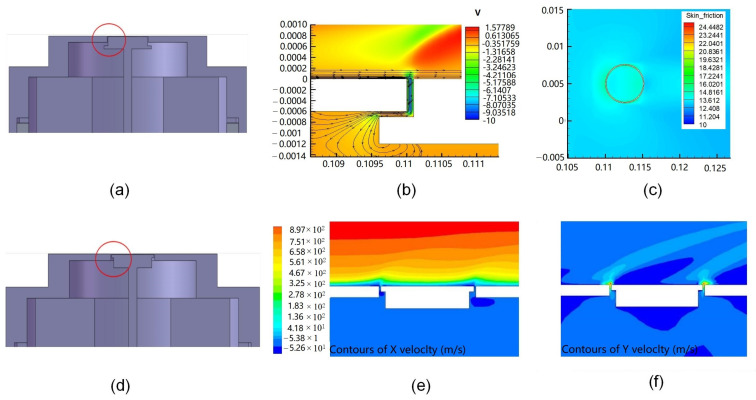
(**a**) The structure of the floating element in our previous work; (**b**) the 2D streamline diagram in the friction sensor; (**c**) the skin friction coefficient of the flat plate; (**d**) the optimized structure of two-layer floating element; (**e**) the velocity distributions in the X direction at floating element under a total pressure of 1.5 MPa; (**f**) the velocity distributions in the Y direction at floating element.

In our previous wind tunnel experiments, the deviations between the measured skin friction coefficients and the analytical values are closed to 100% under laminar flow conditions [10]. The main reason for the error is that the shape of the floating element has an obvious influence on the flow disturbance of the wall fluid, which has an important impact on the friction measurement error. The velocity cloud diagram in the front direction of the floating element of the central section of the friction sensor in the direction of the incoming flow is shown in Figure 3b. It can be seen that the flow field on the surface of the floating element changes due to the suction effect of the moving gap, and the blocking effect of the cylindrical floating element and the diversion effect of the annular gap cause the lateral flow around the cylindrical floating element, and the friction coefficient at the edge of the floating element increases significantly as shown in Figure 3c.

**Figure 4:**
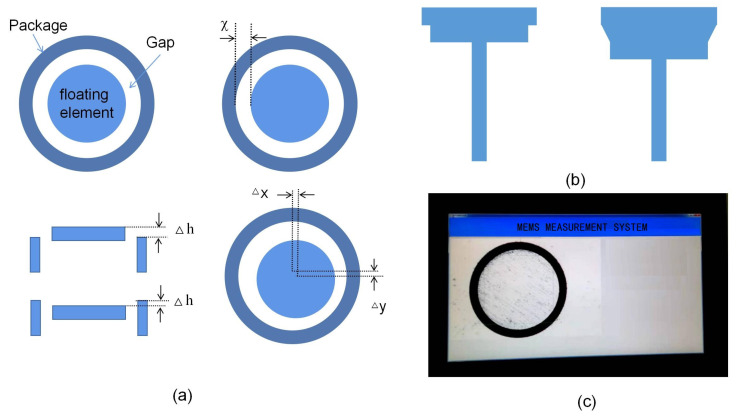
(**a**) The structural error of the prepared sensor; (**b**) the floating element with two-layer structure and three-layer structure; (**c**) the coaxiality measurement of the MEMS sensor.

In order to reduce the influence of the sensor structure on the flow disturbance of the measured wall fluid, the improved floating element is shown in Figure 3d. In addition, the 3D sensor model is also built using CFD, and the velocity distributions in the X direction and Y direction are obtained under a total pressure of 1.5 MPa as shown in Figure 3e,f. The results show that the improved floating element structure can reduce the lateral flow around the cylindrical floating element. A three-layer floating element structure (as shown in Figure 4b) model was also established to further study the influence of floating element structure on the wall flow field and friction measurement error.

The structural error of the prepared sensor mainly includes the gap, misalignment, and non-coaxial between the floating element and the package as shown in Figure 4a. The coaxial assembly problem of the sensor has been solved based on visual alignment technology. The coaxiality measurement of the MEMS sensor is shown in Figure 4c. The position error is only 2 μm, which is about 0.4% of the moving gap with 500 μm. However, the misalignment is difficult to control because the machining accuracy of parts is less than 10 microns. Combined with the wind tunnel experiments results [10] and error analysis results [6,12], the structure appearance of the floating element, the misalignment between the floating element and the package shell, and the gap of the sensor is the main source of measurement error.

**Figure 5:**
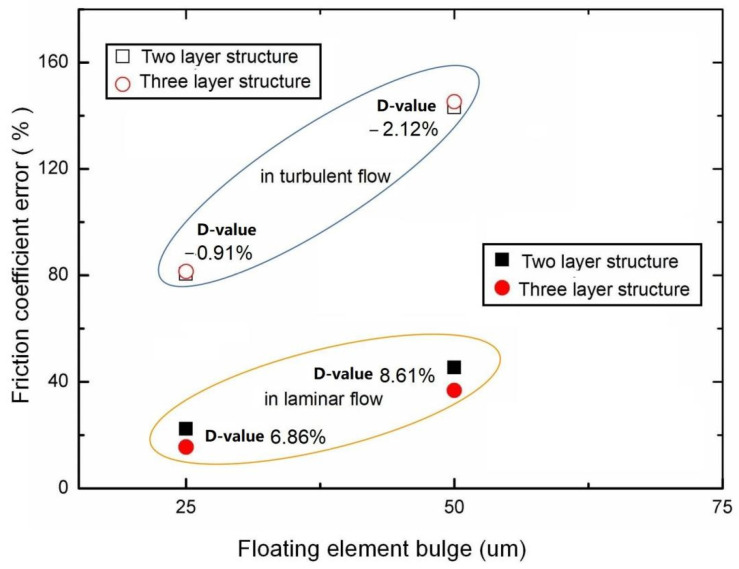
The friction coefficient error of the sensors with a different floating element under different flow fields.

First, the influence of two-layer and three-layer floating elements on the measurement error of friction systems under laminar and turbulent conditions is studied. The diameters of the first and second layers of the floating element are 5 mm and 0.4 mm respectively. The gap of the sensor is 100 μm. The skin friction coefficient of the sensors with different floating elements is shown in Figure 5. When the total pressure of the incoming flow is 0.5 MPa, and the levelness is 25 μm. Compared with the two-layer structure, the friction coefficient error of the three-layer structure is reduced by about 6.86% in laminar flow and increased by 0.91% in a turbulent flow. The main reason for the reduction of the error is that the shape of the floating element of the three-layer structure is obviously weaker than that of the two-layer structure. Meanwhile, the measurement error increases with the increase of the floating element bulge. It also can be seen that the floating element structure has a greater influence on the laminar flow, and the three-layer floating head structure is more conducive to reducing the measurement error.

## 4. Gap Optimization of the MEMS Sensor

The airflow enters the inner cavity of the sensor through the moving gap to deflect the floating element. In the numerical simulation, the friction coefficient directly read out does not take into account the influence of the incoming pressure on the floating element because the floating element cannot rotate, so it is necessary to convert the friction coefficient caused by this part of the pressure. The total friction coefficient includes the surface friction coefficient and the pressure friction coefficient.

**Figure 6:**
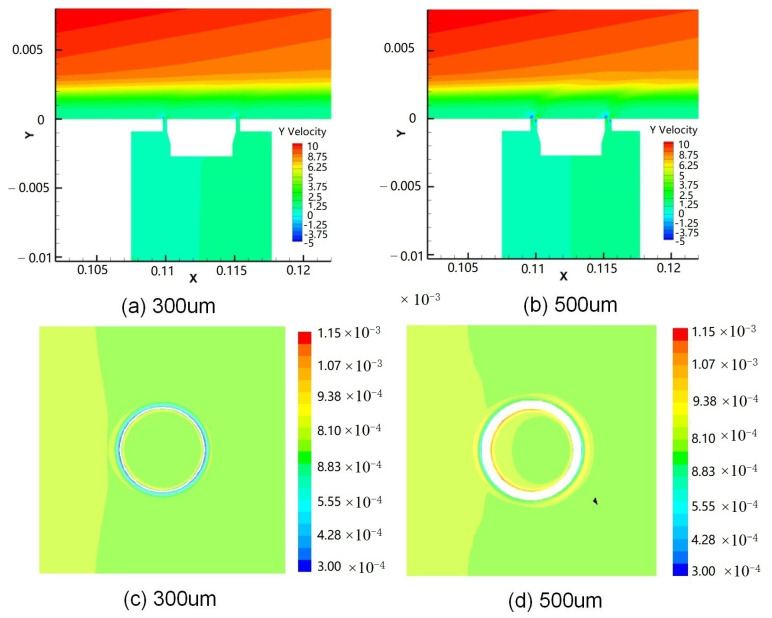
The velocity distributions of the friction sensor with a different gap (**a**) 300 μm and (**b**) 500 μm, and the surface friction coefficient program of the sensor with a different gap (**c**) 300 μm and (**d**) 500 μm.

Ideally, the floating element is perfectly flush with the measuring wall. The velocity distributions of the friction sensor with a different gap in the Y direction under laminar flow are shown in Figure 6a and Figure 6b respectively. It can be seen that the suction effect of the moving gap is enhanced with the increase of the moving gap size, and the flow field on the measuring wall surface is also changed. The surface friction coefficient program of floating elements with different gaps is shown in Figure 6c and Figure 6d respectively. The results show that the direct friction coefficient at the edge of the floating element in the flow direction also increases with the increase of the moving gap size, and the pressure generated by the air flow entering the friction sensor on the floating element decreases with the increase of the gap size, and the gap size has little effect on the total friction coefficient under the condition of complete flush. The total friction coefficient of the sensors with a different gap is shown in Table 2.

Although the moving g has little effect on the error of the total friction coefficient under the condition of complete alignment, it is difficult for the prepared sensor to be completely flush because the machined parts of the sensor always have errors of tens of microns. Combined with the actual assembly error of the MEMS sensor, it is assumed that the protrusion of the floating element is 50 μm. When the floating element is higher than the measuring wall, the effect of the incoming pressure on the floating element becomes obvious, which makes the pressure friction coefficient increase rapidly. At this time, the influence of the gap on the total friction coefficient error increases significantly. The effect of different gaps on the total friction coefficient in laminar flow is shown in Table 3. It can be seen that the direct friction coefficient error changes little with the increase of the moving gap and remains at about 11%. At the same time, the pressure resistance of the incoming flow has an obvious effect on the whole floating element, and the moving gap has a very significant effect on the error of the friction coefficient of the pressure resistance. The pressure friction coefficient error largely determines the total friction coefficient error (the error accounts for 88% of the total error value when the moving gap is 25 μm). Therefore, it is important to control the misalignment of the sensor with a small moving gap to reduce the friction coefficient error caused by pressure.

**Figure 7:**
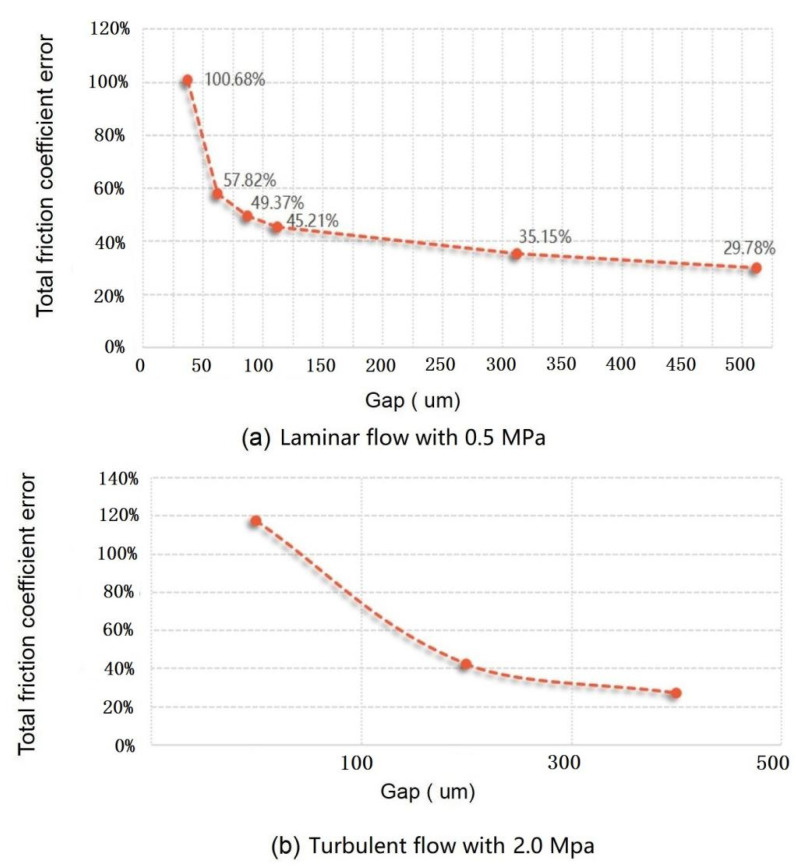
The total friction coefficient error of the sensor with a different gap in laminar flow (**a**) and in turbulent flow (**b**).

The total friction coefficient error of the sensor with a different gap in laminar flow is shown in Figure 7a. It can be seen that the total friction coefficient error exceeds 100% under laminar flow when the gap of the sensor is less than 25 μm, and the total friction coefficient error quickly decreases to 45% with the gap increasing to 100 μm. As the gap increases from 100 to 500, the error only decreases from 45% to 30%, which also shows that when the gap is greater than 300, the impact of the gap on the error becomes weaker, and the impact of the misalignment on the error will be dominant at this time.

The total friction coefficient error of the sensor with a different gap in turbulent flow is shown in Figure 7b. The results show that the total friction coefficient error exceeds 120% under turbulent flow when the gap of the sensor is less than 100 μm, and the error decreases when the gap increases, which is the same as that under laminar flow. As a result, to reduce the measurement error of the friction coefficient, the gap of the sensor should not be less than 500 μm, and it is necessary to minimize the misalignment error at the same time.

## 5. Misalignment Optimization of the MEMS Sensor

The structure of MEMS skin friction is composed of a silicon differential-capacitor embedded with a floating element, signal readout circuit, and package metal shell. The machining accuracy of parts such as floating elements and package metal shells is about 10 microns. Therefore, the total misalignment error is usually in the range of −50 μm to 50 μm.

**Figure 8:**
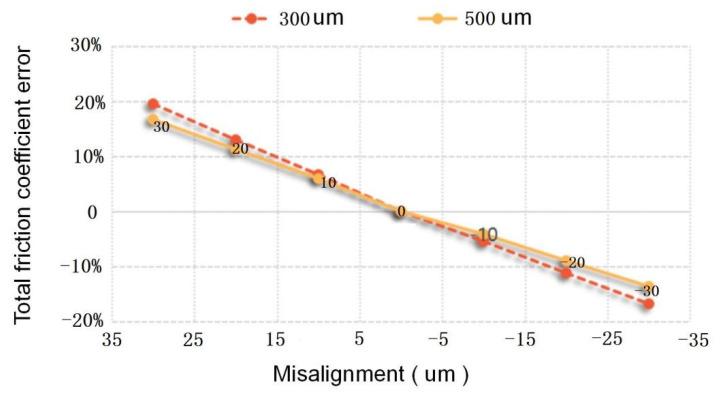
Influence of different misalignment on friction measurement error in laminar low.

The influence of different misalignment on friction measurement error is analyzed, and the total friction coefficient error of the sensor with different misalignment in laminar flow (0.5 MPa) is shown in Figure 8. The results show that the influence of the convex of the floating element on the friction coefficient is higher than that of the concave of the floating element, and the friction measurement error decreases with the increase of the gap. Therefore, it is necessary to ensure that the floating element is lower than the measuring wall surface to avoid reverse steps affecting the flow field and increasing the measurement error. In addition, the misalignment error is reduced to 20 μm by selecting parts and controlling the adhesive thickness in practice.

## 6. Conclusions

Due to the components of the sensor including MEMS chip, floating element, signal readout circuit, and package shell being fabricated and assembled by using various processes, the structural error of the prepared sensor including the gap, misalignment, and non-coaxial between the floating element and the package is inevitable. Those structure errors of the sensor including the protrusion of the sensing element and the measurement gap will change the characteristics of the fluid, which will seriously affect the accuracy of the sensor in measuring the skin friction resistance. This paper developed a 3D numerical model of MEMS skin friction sensor in the hypersonic flow field based on CFD to research the influence of sensor-sensitive structure on wall-flow characteristics and friction perceptual characteristics. The numerical simulation method and calculation model constructed are effective, which is verified by using the Blathius solution of two-dimensional laminar flow on a flat plate, and the error is less than 0.4%. In addition, to improve the measurement accuracy of skin friction, the design criterion of the sensor-sensitive unit structure is obtained as follows:

(1) Compared with the turbulent flow, the three-layer structure of the floating head has a greater impact on the laminar flow, and the three-layer structure of the floating head is better than the two-layer structure.
(2) The moving gap of the floating element in the friction sensor has a great impact on the friction measurement. Under the same alignment, the friction coefficient error decreases with the increase of the moving gap, so the moving gap should be appropriately increased.
(3) The influence of the floating element misalignment on the friction measurement is significantly reduced with the increase of the clearance. In practice, it is necessary to ensure that the floating element is lower than the measuring wall surface to avoid reverse steps affecting the flow field and increasing the measurement error.

Then, based on the above rules, the friction coefficient measurement error of the MEMS sensor with a three-layer floating element structure, a 500 μm moving gap, and the floating element misalignment between 10 μm and −20 μm is better than 10%. The future work is to prepare the sensor with special parameters and design the corresponding experimental scheme to verify the effectiveness of the model.

## Figures and Tables

**Table 1:** The main parameters of the wind tunnel.

Nominal Mach Number (*M*_∞_)	Total Pressure *P*_0_ (M Pa)	Total Temperature *T*_0_ (K)	Static Pressure *P*_∞_ (Pa)	Dynamic Pressure *q*_∞_ (Pa)	Unit Reynolds Number *Re*/*L* (L/m)
6	0.5	417	316.68	7980.4	5.679 × 10^6^
	1.0	437	633.33	15961	1.053 × 10^7^
	1.5	449	950.04	23941	1.506 × 10^7^
	2.0	458	1266.7	31924	1.941 × 10^7^
	2.5	466	1583.4	39902	2.361 × 10^7^

**Table 2:** The total friction coefficient of the sensors with a different gap in laminar flow.

Gap (μm)	Misalignment (μm)	Friction Coefficient Reading Value	Pressure Friction Coefficient	Total Friction Coefficient
300	0	7.5613 × 10^−4^	8.6533 × 10^−6^	7.6478 × 10^−4^
500	0	7.5648 × 10^−4^	8.0592 × 10^−6^	7.6454 × 10^−4^

**Table 3:** Effect of different gaps on total friction coefficient in laminar flow.

Gap (μm)	Friction Coefficient Reading Value	Pressure Friction Coefficient	Total Friction Coefficient	Friction Coefficient Reading Value Error	Pressure Friction Coefficient Error	Total Friction Coefficient Error
25	8.4706 × 10^−^^4^	6.6998 × 10^−^^4^	1.5170 × 10^−^^4^	12.05%	88.63%	100.68%
50	8.4608 × 10^−^^4^	3.4693 × 10^−^^4^	1.1930 × 10^−^^4^	11.92%	45.89%	57.82%
75	8.4576 × 10^−^^4^	2.8340 × 10^−^^4^	1.1292 × 10^−^^4^	11.88%	37.49%	49.37%
100	8.4513 × 10^−^^4^	2.5258 × 10^−^^4^	1.0977 × 10^−^^4^	11.80%	33.41%	45.21%
300	8.4171 × 10^−^^4^	1.7997 × 10^−^^4^	1.0217 × 10^−^^4^	11.34%	23.81%	35.15%
500	8.3676 × 10^−^^4^	1.4434 × 10^−^^4^	9.8110 × 10^−^^4^	10.69%	19.09%	29.78%

## Data Availability

Data are contained within the article.
